# Association between V̇O_2max_, handgrip strength, and musculoskeletal pain among construction and health care workers

**DOI:** 10.1186/s12889-017-4173-3

**Published:** 2017-03-21

**Authors:** Lene Lehmann Moberg, Lars-Kristian Lunde, Markus Koch, Anne Therese Tveter, Kaj Bo Veiersted

**Affiliations:** 1 National Institute of Occupational Health, PO Box 8149 Dep, 0033 Oslo, Norway; 2 Faculty of Health Sciences, Oslo and Akershus University College of Applied Sciences, PO Box 4, St. Olavsplass, 0130 Oslo, Norway

**Keywords:** Musculoskeletal disorders, Physical fitness, Aerobic capacity, Muscular strength, Health care, Construction

## Abstract

**Background:**

Construction and health care workers have a high prevalence of musculoskeletal disorders, and they are assumed to have physically demanding jobs. Profession- and gender-specific associations between individual capacity and musculoskeletal pain have not been sufficiently investigated. The main aim of this study was to examine the association between individual capacity (maximal oxygen uptake (V̇O_2max_) and handgrip strength) and musculoskeletal pain among construction and health care workers.

**Methods:**

This cross-sectional study examined 137 construction and health care workers (58 women and 79 men) with a mean age of 41.8 years (standard deviation 12). Aerobic capacity was indirectly assessed by the Åstrand cycle test, and strength was assessed by a handgrip test. Musculoskeletal pain was described by total pain, divided into neck, shoulder, and low back pain, during the last 12 months, and it was dichotomized in below or above 30 days. Logistic regression was used to analyse the associations between V̇O_2max_, strength, and musculoskeletal pain in the total study sample and separately for construction and health care workers. Analyses were adjusted for age, gender, body mass index (BMI), and selected mechanical and psychosocial factors.

**Results:**

Every second participant (51.8%) reported pain in either neck, shoulders or low back for more than 30 days during the last 12 months. Among the health care workers, a small but significant association was found between a high V̇O_2max_, high handgrip strength, and a low level of musculoskeletal pain. No association was found for the construction workers.

**Conclusions:**

An association between V̇O_2max,_ handgrip strength, and musculoskeletal pain was found for health care workers but not for construction workers. These results indicate that activities promoting individual capacity may reduce musculoskeletal pain for health care workers.

**Electronic supplementary material:**

The online version of this article (doi:10.1186/s12889-017-4173-3) contains supplementary material, which is available to authorized users.

## Background

Musculoskeletal disorders (MSDs) are a major concern in the European population and constitute one of the main reasons for individual complaints and sickness absences [1]. The most prevalent work-related MSDs are low back pain and neck and shoulder pain [2]. The construction and health care sectors have a high prevalence of MSDs [3, 4] and are the sectors with the highest sickness absence rates in Norway [5]. Risk factors for developing work-related MSDs are linked to both mechanical and psychosocial exposures [6]. Construction and health care workers are assumed to have physically demanding jobs and to be exposed to work requiring high energy metabolism and considerable muscle force. A gap between work demand and an individual’s capacities of maximal oxygen uptake and muscular strength may increase the risk of MSDs [7]. To reduce this gap, physical activity worksite programmes have been offered to employees, but there are conflicting results regarding the efficacy of such programmes with respect to musculoskeletal pain [8]. An effect of these interventions presupposes a relationship between individual capacity and musculoskeletal pain or between physical activity and musculoskeletal pain. In the present study we therefore hypothesize a negative association between fitness, strength and MSDs.

A review by the Swedish Work Environment Authority summarized the research on physical activity and physical capacity in relation to work-related MSDs. The majority of the included studies were based on self-reported physical activity. The results were contradictory but indicated that a high level of physical activity may reduce work-related MSDs. Only eight of the included studies described the relationship between physical capacity and musculoskeletal pain, and the results were inconclusive. These inconclusive results revealed a lack of knowledge regarding the association between measured physical capacity and work-related MSDs. According to this review, few studies had separated the results according to profession or gender. The authors suggested that future research should focus on gender and occupational differences [8].

The main aim of this study was to explore the association between individual capacity (maximal oxygen uptake (V̇O_2max_) and handgrip strength) and musculoskeletal pain among construction and health care workers. Additional aims were to analyse possible occupational differences in this association, and to relate self-reported physical activity to musculoskeletal pain and V̇O_2max_.

## Methods

### Study design and subjects

This study was designed as part of a larger longitudinal cohort study in the construction and health care sectors [9]. Baseline data were collected from the 2^nd^ quarter of 2014 to the 2^nd^ quarter of 2015. The present paper is a cross-sectional study of selected baseline data.

**Fig. 1:**
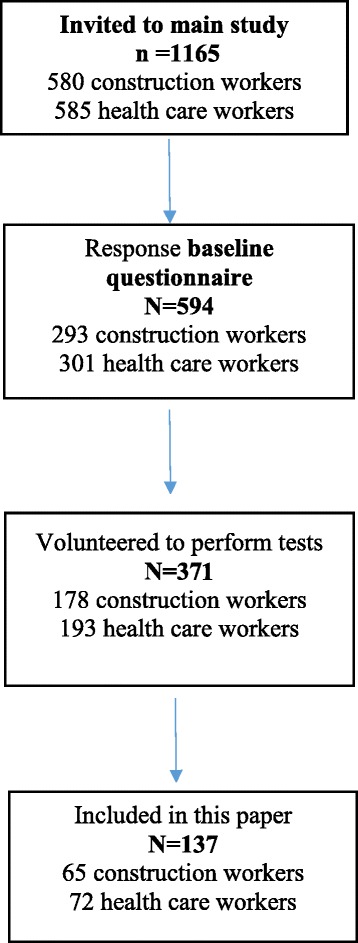
Flow-chart of subjects (for comments see text)

Participants were recruited from four construction enterprises and a group of municipality nursing homes and home care units. Inclusion criterion was adequate skills in reading and writing Norwegian. A total of 580 construction workers and 585 health care workers were invited, of which 50.5 and 51.5%, respectively, answered the baseline questionnaire. See Fig. 1 for subject flow-chart. Sixty-five construction workers and 72 health care workers were included in this study, based on willingness to perform tests, availability and representative distribution of different occupations. Exclusion criteria were cardiovascular disease or considerable musculoskeletal pain on the test day. For background variables see Table 1.

**Table 1:** Background characteristics, exposure and outcome measures of the study population stratified by occupational sector

	Total sample (*n* = 137)		Construction (*n* = 65)		Health care (*n* = 72)		*P*-value^*^
	N (%)	Mean (SD)	N (%)	Mean (SD)	N (%)	Mean (SD)	
Gender, (female)	58(42.3)		1(1.5)		57(79.2)		<0.001
Age, (years)		41.8(12)		40(13.2)		42.3(10.8)	0.086
BMI, (kg/m^2^)		25.5(3.8)		25.6(3.4)		25.5(4.2)	0.379
V̇O_2max_, (ml/min/kg)*, n = 126*		34.9(10.5)		37.8(10.5)		32.2(9.9)	<0.001
Strength (kg), *median/range*		43.5(24–77)		53(28–77)		33.5(24–67)	<0.001
Musculoskeletal pain (>30d/y)							
Total	71(51.8)		32(49.2)		39(54.2)		0.564
Low back	37(27)		14(21.5)		23(31.9)		0.171
Neck	31(22.6)		9(13.8)		22(30.6)		0.020
Shoulders	53(38.7)		24(36.9)		29(40.3)		0.639
Part of working day with lifting in awkward positions							0.787
Very little/not at all	91(66.4)		40(61.5)		51(70.8)		
¼	31(22.6)		16(24.6)		15(20.8)		
½	9(6.6)		4(6.2)		5(6.9)		
¾	4(2.9)		3(4.6)		1(1.4)		
Almost all time	2(1.5)		2(3.1)		0(0)		
Supportive and encouraging							0.578
leader and colleagues							
Very little/not at all	2(1.5)		1(1.5)		1(1.4)		
Fairly small	5(3.7)		1(1.5)		4(5.7)		
Somewhat	23(17)		14(21.5)		9(12.9)		
Fairly much	75(55.6)		40(61.5)		35(50)		
Very much	30(22.2)		9(13.8)		12(30)		
Smoking (yes)	39(28.3)		20(30.8)		19(26.4)		0.570
Light physical activity (hours per week)							0.399
0	6 (4.3)		3 (4.6)		3 (4.2)		
Less than 1	14 (10.1)		4 (6.2)		10(13.9)		
1–2	36 (26.1)		20 (30.8)		16 (22.2)		
3 or more	61 (44.2)		29 (44.6)		32 (44.4)		
Hard physical activity (hours per week)							0.677
0	13 (9.4)		7 (10.8)		6 (8.3)		
Less than 1	26 (18.8)		10 (15.4)		16 (22.2)		
1–2	42 (30.4)		18 (27.7)		24 (33.3)		
3 or more	39 (28.3)		20 (30.8)		19 (26.4)		

^*^Tests for statistical significance comparing construction and health care workers were performed with Fisher’s exact test, Pearson Chi-square and Mann-Whitney *U* test when appropriate

### Measures

#### Musculoskeletal pain

Musculoskeletal pain was reported by use of the Standardized Nordic Questionnaire [10] on the same day as the individual capacity tests. In this study, focus was on the duration of pain in the neck, shoulders, and low back. Participants responded to questions with five response options: 0 days, 1–7 days, 8–30 days, more than 30 days (but not daily) and daily pain during the last 12 months. The pain duration variables were dichotomized in 30 days or less and more than 30 days. Thirty days as the cut-point has been used in previous studies [11, 12].

#### V̇O_2max_

Aerobic capacity was determined using a submaximal Åstrand ergometer test (Ergometer 839 E, Varbeg, Sweden) [13]. According to standard guidelines, subjects pedalled at a frequency of 50 revolutions per minute at an external power between 75 and 150 watts. The test was terminated when the subject’s heart rate was stable and greater than 120 beats per minute, which normally occurred between the 5^th^ and 6^th^ minute. The mean steady-state heart rate was used to estimate V̇O_2max_ based on the Åstrand monogram [14], modified for age and gender [15]. The Åstrand monogram had been validated for clinical monitoring and research purposes [16] and had previously been used for the estimation of aerobic capacity among workers [17–19].

#### Handgrip strength

Maximal isometric handgrip strength was determined using a hand dynamometer (Lafayette Instrument, Indiana, USA) according to standard procedures [20]. The maximum value of three attempts was used in the analysis. The average of the right and left hand was chosen as the handgrip strength. The handgrip test was chosen because it is indicative of general muscular strength [21, 22] and is a practical and efficient test to measure a worker’s strength. Additionally, a hand dynamometer was easy to transport, and the tests were quick and easy to perform.

#### Self-reported physical activity

Information regarding physical activity was obtained from the baseline questionnaire. Participants were asked to provide the average of their weekly hours of light (not sweating and panting) and hard (sweating and panting) activity during the last 12 months. They had four response options for both questions: No, less than 1, 1–2 or ≥ 3 h per week.

#### Confounders

A systematic review has revealed associations between age, BMI, gender, mechanical and psychosocial factors and musculoskeletal pain [6]. Therefore, we adjusted for these factors in the analyses. Information on age, gender, body mass index (BMI), and selected mechanical and psychosocial factors was based on self-reported data. BMI was calculated using weight and height (kg/m^2^). Psychosocial factors were determined from validated questionnaires on psychosocial and organizational factors [23]. Participants were asked how encouraging and supportive they perceived their colleagues and leaders to be. The response options were the following: very little or not at all, fairly small, somewhat, fairly much, very much. Participants were also asked if they were lifting in awkward positions [24]. They had two response options, “yes” or “no”. If they answered “yes”, they were asked to select one of the five response options to indicate the proportion of the working day that they lifted in these positions: Very little, ¼ of the working day, ½ of the working day, ¾ of the working day or almost all the time. The two first response options (no or very little) were merged and given the new name “very little, not at all”.

### Statistical analysis

Data was checked for normality and outliers, as background for statistical approach. Differences between the groups of construction and health care workers were analysed with independent samples t-tests or Mann-Whitney U tests. Multivariate logistic regression models were used to explore the association between V̇O_2max_, strength, self-reported physical activity and musculoskeletal pain. These models were analysed with either V̇O_2max_, strength or physical activity as predictor. Odds ratio (OR) with confidence interval (95% CI) was used as risk estimate, meaning that a step change of predictor unit changed risk according to the OR value. The outcome variables were total musculoskeletal pain and pain separated for the neck, shoulders and low back. To survey possible occupational differences in the association between V̇O_2max_, handgrip strength, and musculoskeletal pain, we also performed separate analyses for the construction and health care workers.

The analyses were adjusted for age, gender, BMI, and selected mechanical and psychosocial factors. To limit the number of variables in the logistic regression, analyses were not adjusted for other confounders. Information on smoking was removed from the multivariate analyses since inclusion of the variable did not change any of the results.

The correlation between self-reported physical activity and measured V̇O_2max_ was examined by Spearman correlation analysis.

Data were analysed using IBM SPSS version 22 (IBM Corporation, USA). A *p*-value < 0.05 was used as significance level in the analyses.

## Results

### Musculoskeletal pain

Considerable musculoskeletal pain was reported in both occupational groups. As shown in Table 1, 49.2% of the construction workers and 54.2% of the health care workers reported pain in the low back, neck, or shoulders for more than 30 days during the last year.

### Differences between groups

There were differences in gender, neck pain, V̇O_2max_, and handgrip strength between the construction workers and health care workers (Table 1). The mean V̇O_2max_ was 37.7 ml/min/kg (SD: 10.5) among the construction workers, compared with 32.2 ml/min/kg (SD: 9.9) among the health care workers. The median value of the handgrip strength was 53 kg (range: 28.5–77 kg) among the construction workers and 33.5 kg (range: 24–67 kg) among the health care workers. There were no differences in self-reported light (*p* = 0.748) or hard (*p* = 0.598) physical activity between the groups.

### Associations between V̇O_2max_, strength, and musculoskeletal pain

#### Total study sample

Several associations between V̇O_2max_ and musculoskeletal pain and between strength and musculoskeletal pain were statistically significant in the unadjusted analysis (Table 2). After adjusting for age, gender, BMI, and selected mechanical and psychosocial factors, only the association between handgrip strength and low back pain remained statistically significant. No association was found between V̇O_2max_ and low back pain (*p* = 0.056) in the adjusted analysis. Age (odds ratio [OR] = 1.05, 95% confidence interval [CI] 1.01, 1.09, *p* =0.008) and lifting in awkward positions (OR = 1.95, 95% CI: 1.19, 3.19, *p* = 0.008) were associated with total musculoskeletal pain in the multivariate regression analysis.

**Table 2:** Total study sample, *n* = 137. VO2max (ml/min/kg, *N* = 122) and strength (kg, *N* = 131) associated with musculoskeletal pain. Unadjusted and adjusted^a^ odds ratios (OR) with confidence intervals (95% CI) and *p*-values

	Unadjusted		Adjusted^a^	
	OR (95% CI)	*P*-value	OR (95% CI)	*P*-value
Total pain				
VO2max	0.95 (0.92, 0.99)	0.018	0.98 (0.93, 1.02)	0.280
Strength	0.97 (0.94, 0.99)	0.024	0.96 (0.91, 1.06)	0.160
Low back pain				
VO2max	0.94 (0.89, 0.99)	0.015	0.94 (0.88, 1.00)	0.056
Strength	0.96 (0.93, 0.99)	0.014	0.92 (0.86, 0.98)	0.008
Neck pain				
VO2max	1.00 (0.96, 1.04)	0.842	1.00 (0.96, 1.06)	0.723
Strength	0.94 (0.91, 0,98)	0.003	0.96 (0.90, 1.03)	0.262
Shoulder pain				
VO2max	0.96 (0.92, 1.00)	0.052	0.98 (0.94, 1.03)	0.408
Strength	0.98 (0.95, 1.01)	0.098	1.00 (0.95, 1.06)	0.955

^a^Adjusted for gender, age, BMI, selected psychosocial and mechanical factors

#### Construction workers

No association was found between V̇O_2max_, strength, and musculoskeletal pain for the construction workers (Table 3). The multivariate regression analysis showed that age (OR = 1.08, 95% CI: 1.02, 1.15, *p* = 0.006) and lifting in awkward positions (OR = 1.89, 95% CI: 1.0, 3.57, *p* = 0.049) were the only variables that were associated with musculoskeletal pain in this group.

**Table 3:** Construction workers, *n* = 65. VO2max (ml/min/kg, *N* = 59) and strength (kg, *N* = 63) associated with musculoskeletal pain. Unadjusted and adjusted^a^ odds ratios (OR) with confidence intervals (95% CI) and *p*-values

	Unadjusted		Adjusted^a^	
	OR (95% CI)	*P*-value	OR (95% CI)	*P*-value
Total pain				
VO2max	0.99 (0.94, 1.04)	0.576	1.02 (0.96, 1.09)	0.473
Strength	0.99 (0.94, 1.04)	0.681	1.04 (0.96, 1.13)	0.366
Low back pain				
VO2max	0.96 (0.89, 1.04)	0.326	1.00 (0.92, 1.10)	0.940
Strength	0.95 (0.89, 1.02)	0.156	0.92 (0.83, 1.02)	0.116
Neck pain				
VO2max	1.06 (0.99, 1.12)	0.100	1.08 (0.99, 1.17)	0.056
Strength	1.03 (0.95, 1.12)	0.435	1.04 (0.95, 1.15)	0.387
Shoulder pain				
VO2max	0.98 (0.93, 1.04)	0.550	1.02 (0.96, 1.08)	0.575
Strength	1.01 (0.95, 1.07)	0.814	1.08 (0.99, 1.18)	0.077

^a^Adjusted for gender, age, BMI, selected psychosocial and mechanical factors

Approximately 20% of the construction workers were office workers. The analyses above were also performed by excluding data for the office workers, which did not change the results.

#### Health care workers

Multivariate regression analyses showed an association between V̇O_2max_ and musculoskeletal pain for the health care workers (Table 4). For every one-unit ml/min/kg V̇O_2max_ increase, the likelihood that the health care workers would report musculoskeletal pain decreased by approximately 10%. The relationship between V̇O_2max_ and low back pain showed the strongest association.

**Table 4:** Health care workers, *n* = 72. VO2max (ml/min/kg, *N* = 63) and strength (kg, *N* = 68) associated with musculoskeletal pain. Unadjusted and adjusted^a^ odds ratios (OR) with confidence intervals (95% CI) and *p*-values

	Unadjusted		Adjusted^a^	
	OR (95% CI)	*P*-value	OR (95% CI)	*P*-value
Total pain				
VO2max	0.91 (0.84, 0.98)	0.008	0.90 (0.82, 0.99)	0.026
Strength	0.93 (0.87, 0.98)	0.009	0.88 (0.80, 0.97)	0.010
Low back pain				
VO2max	0.92 (0.85, 0.99)	0.039	0.88 (0.79, 0.98)	0.022
Strength	0.96 (0.90, 1.01)	0.132	0.91 (0.82, 0.99)	0.039
Neck pain				
VO2max	0.98 (0.93, 1.04)	0.531	0.95 (0.87, 1.03)	0.170
Strength	0.88 (0.80, 0.97)	0.008	0.85 (0.75, 0.96)	0.011
Shoulder pain				
VO2max	0.93 (0.87, 0.99)	0.043	0.91 (0.83, 0.99)	0.039
Strength	0.93 (0.87, 0.99)	0.022	0.91 (0.83, 1.00)	0.057

^a^Adjusted for gender, age, BMI, selected psychosocial and mechanical factors

Handgrip strength was also associated with musculoskeletal pain in this group. An increase of one kg in handgrip strength decreased the probability of reporting musculoskeletal pain by approximately 12%. Strength was associated with low back pain and neck pain, separately.

The multivariate regression analysis also showed that lifting in awkward positions (OR = 2.86, 95% CI: 1.14, 7.22, *p* = 0.026) was associated with low back pain in this group.

### Self-reported physical activity

Both self-reported light and hard physical activity were associated with total musculoskeletal pain for the health care workers, but not for the construction workers (Table 5). E.g. an increase from less than 1 h to 1–2 h per week of self-reported activity may reduce musculoskeletal pain for health care workers by approximately 50%.

**Table 5:** Light and hard physical activity (0–3) associated with total musculoskeletal pain. Unadjusted and adjusted^a^ odds ratios (OR) with confidence intervals (95% CI) and *p*-values (*N* = 137)

	Total study sample		Construction		Health care	
	OR (95% CI)	*P*-value	OR (95% CI)	*P*-value	OR (95% CI)	*P*-value
Light physical activity	0.50 (0.29, 0.87)	0.015	0.46 (0.19, 1.13)	0.091	0.47 (0.22, 0.99)	0.048
Hard physical activity	1.36 (0.60, 3.07)	0.467	0.96 (0.48, 1.90)	0.905	0.52 (0.28, 0.99)	0.045

^a^Adjusted for gender, age, BMI, selected psychosocial and mechanical factors

Hard physical activity was positively correlated with V̇O_2max_ (*r* = 0.3; *p* = 0.001) for the total study sample, and light physical activity was not (*p* = 0.065).

## Discussion

This study aimed to examine the associations between individual capacity (V̇O_2max_ and handgrip strength) and musculoskeletal pain in construction and health care workers. Every second participant (51.8%) reported pain in either neck, shoulders or low back for more than 30 days during the last 12 months. The associations between individual capacity (V̇O_2max_ and handgrip strength) and musculoskeletal pain showed occupational differences; specifically, increased individual capacity was associated to lower musculoskeletal pain for health care workers but not for construction workers.

Previous studies on health care workers have examined the associations between physical capacity or physical activity and musculoskeletal pain. One study concluded that self-assessed low physical capacity was a strong predictor for developing non-chronic and persistent low back pain among health care workers [12]. Other studies showed associations between the level of physical activity and work ability [25] and between the level of physical activity and sickness absence among health care workers [26]. These studies did not include V̇O_2max_ or strength tests in their analyses, but the conclusions were in accordance with those from our study. In contrast, one study examined risk factors for low back pain among female nursing personnel and revealed that low self-assessed fitness and low physical activity were not among the risk factors [27]. Intervention studies may give some information on the effect of activity or changed individual capacity on risk for musculoskeletal disorders, but these studies have presented conflicting results. One intervention study that aimed at reducing musculoskeletal pain among health care workers showed positive effects of Zumba and football exercise on neck and shoulder pain, but not on low back pain [28]. Another study of lifestyle interventions among health care workers did not find effect on musculoskeletal pain [29]. These studies on health care workers had different design and measurements, which may explain the different results.

An exercise intervention designed to improve aerobic capacity among construction workers showed no effect on musculoskeletal pain [19]. Strength training for this occupational group showed no effect on shoulder pain [30]. An exploratory study in construction workers, which included self-reported physical fitness in the analyses, did not mention physical fitness among the factors related to musculoskeletal pain [31]. The results from our study confirmed the results from these studies. The lack of association between individual capacity and musculoskeletal pain among construction workers found in our study, may be due to larger effect on pain of other risk factors such as awkward work postures. This may also explain the lack of an effect of exercise interventions in this group.

The two sectors were gender-segregated, and it was not possible to distinguish between gender and profession. The results showing occupational differences may have exposed gender differences as well as occupational differences. The review from the Swedish Work Environment Authority on work-related MSDs and physical activity pointed out the lack of previous research focusing on gender differences, but it showed that the association between physical activity and musculoskeletal pain was more clear in studies that only included women [8]. The results from our study support this finding. Our results from the analysis with low back pain as the outcome variable can be compared with those of the aforementioned study in female health care workers, as that study revealed an association between low physical capacity and increased low back pain [12]. An association was found in our study between low back pain and individual capacity in mainly female health care workers, but not among male construction workers. A 6.5-year follow-up study in young adults examined the association between leisure time physical activity and low back pain. Even though that study did not reveal a clear relationship between physical activity and low back pain, a trend was found in females [32]. Contrary to this gender-related association, increased aerobic fitness among male conscripts aged 18–19 years was moderately associated with a reduced risk of non-injury musculoskeletal sickness absence 5–15 years later [33]. Previous gender-segregated studies were not conclusive.

To understand the different results in the two occupational groups, we explored the different characteristics between the groups. The health care workers had a significantly lower V̇O_2max_ and strength than that of the construction workers, which was predicted because women’s maximal aerobic capacity and muscular strength are lower than those of men [2, 34]. A conceptual model that included exposure, dose, capacity, and response illustrated a possible mechanism between low levels of muscular strength and musculoskeletal pain [7]. Exposure referred to external factors, in this context, work requiring considerable muscle force. The ability to “resist” doses of this exposure is determined by the muscle strength capacity. A high load repeated over time may lead to tissue damage, inflammation of the musculoskeletal tissue, and pain. These changes may result in reduced capacity and, in this context, reduced pain-dependent strength [7]. An adaption of the musculoskeletal tissue presupposes a mechanical exposure and recovery that are customized to the individual. Individuals with low muscular strength use a greater proportion of their maximum capacity than individuals with greater strength to perform the same work and will reach an assumed level of risk for tissue damage and pain faster [2]. The same model may illustrate the mechanism involved in the association between the level of VO_2max_ and musculoskeletal pain. The ability to manage work demanding a high energy metabolism may be determined by the aerobic capacity [2]. The model only fit our results for health care workers. With a lower V̇O2_max_ and strength, the gap between work demand and individual capacity seemed to be larger for health care workers than construction workers. A low capacity may lead to an increased risk of pain development, and a higher capacity may be a possible modifier. This relationship was not found for construction workers, in whom other work-related factors were more strongly associated with musculoskeletal pain.

Genetic contributions to individual capacity are important, but aerobic capacity and muscular strength are largely determined by the level of physical activity [35]. Physical activity is assumed to have an independent preventive effect on musculoskeletal pain. We also found a significant association between self-reported physical activity and musculoskeletal pain in health care workers but not in construction workers, although there were no significant differences in self-reported physical activity between the groups. Previous research had shown that self-reported physical activity and measured V̇O_2max_ were correlated but that the correlation was low [36] or moderate [37]. These results were supported by our data. The moderate correlation may be due to the limitations associated with self-reports [38], as participants may have had trouble remembering and averaging values of physical activity 1 year previously. Another possible explanation is that the levels of V̇O_2max_ and strength were the central factors associated with musculoskeletal pain rather than physical activity. A study in a working population supported this explanation and concluded that physical fitness showed a higher association with low back pain compared to self-reported physical activity. The authors suggested that for the prevention of pain, physical activity should be performed at an intensity and at a duration that contribute to an increased V̇O_2max_ [39].

Exercise programs at the workplace initiated to reduce musculoskeletal pain showed mixed results [8]. Exercise may not reduce symptoms for all workers with musculoskeletal pain, but the results from our study may help identify the groups to be focused upon. Our results indicate that activities promoting individual capacity may reduce musculoskeletal pain for health care workers.

### Strengths and limitations

The strength of this study was the assessment of both objectively measured and self-reported data from two different sectors. Aerobic capacity and strength were objectively measured, which was a strength compared with the utilization of self-reported physical fitness or physical activity data. V̇O_2max_ was calculated indirectly using the Åstrand test. The validity and reliability of the submaximal Åstrand test have been documented [16], but the results are less accurate than laboratory tested V̇O_2max_. A direct V̇O_2max_ test is extremely demanding for the participant and may compromise work activities. Portable equipment made the Åstrand test practical for testing workers during working hours, and the submaximal test was more comfortable for workers to perform. A limitation may be random deviations from the estimated maximal heart rate, which can affect the individual results. However, with the number of participants used in this study, this limitation will probably be of minor importance. The process used to select patients to perform the test may have influenced the results, as only the participants who accepted participation could be selected. Individuals diagnosed with cardiovascular disease and those who had considerable musculoskeletal pain were excluded because they were not permitted to perform the tests. These criteria may have excluded the participants with the lowest aerobic capacity and those with the highest level of pain, however, very few were excluded for this reason. Handgrip strength was used as an indicator of general strength. This association has been well documented previously [21] and was, therefore, viewed as appropriate in this study. The availability of information on self-reported mechanical and psychosocial exposures provided a good opportunity to adjust for confounding variables.

This study has a small sample size and the power of the study is therefore limited and results have to be interpreted cautiously. This was a cross-sectional study, and it was therefore possible that musculoskeletal pain may influence the individual capacity parameters. The ergometer test may provoke back pain and the handgrip strength test may provoke shoulder/neck pain. However, the pain questions the same day as measurements were dichotomized in above or below 30 days duration the last year, so these possible acute responses should not be influenced by the tests. The cross-sectional design of the study make it impossible to evaluate a potentially causal effect of improved V̇O_2max_ and strength related to musculoskeletal pain.

To limit the number of variables in the logistic regression, the analyses were not adjusted for smoking. The chosen mechanical and psychosocial exposure variables were not exhaustive but were selected from the set of variables assessed in the questionnaire. Self-reported pain during the previous year was used as a measure of musculoskeletal pain. It may be difficult to recall pain during the previous year, and responses may be influenced by the reporting style [40]. Dichotomisation of data may be a limitation in regression models. However, we dichotomised number of days with pain to distinguish between a few days during the last year and substantial number of days with pain among workers.

## Conclusions

This study revealed occupational differences in the association between V̇O_2max_, handgrip strength, and musculoskeletal pain. Small but statistical significant associations were found for health care workers but not for construction workers. Due to the gender-segregated sectors included in this study, it was not clear whether the differences were related to occupation or gender. Increased levels of V̇O_2max_ and strength were associated with less musculoskeletal pain in health care workers, which indicate that activities promoting higher V̇O_2max_ and hand strength may reduce musculoskeletal pain in this group. However, this is a small study and results should be interpreted cautiously. Further research with a similar design but larger samples is needed to determine whether there are occupational and gender differences in the association between V̇O_2max_, strength, and musculoskeletal pain.

## Additional file

Additional file 1: All data Norwegian Moberg Veiersted. (XLSX 198 kb)

## Abbreviations

BMI: Body mass index
CI: Confidence interval
MSD: Musculoskeletal disorders
OR: Odds ratio
SD: Standard deviation
SPSS: Statistical package for the social sciences
V̇O_2max_: Maximal oxygen uptake
